# Anxiety and depression among breast cancer patients

**DOI:** 10.1192/j.eurpsy.2023.2001

**Published:** 2023-07-19

**Authors:** F. Zaouali, I. Abbes, A. Ben Slama, I. Belhaj Youssef

**Affiliations:** 1 Outpatient Medical Oncology consultation, Haj Ali Soua regional hospital, Ksar Hellal, Monastir; 2 Family Medicine Department, University of Monastir, Monastir; 3 Outpatient Physical Medicine and Rehabilitation consultation, Haj Ali Soua regional hospital, Ksar Hellal, Monastir, Tunisia

## Abstract

**Introduction:**

Neoplastic disease affects all aspects of life. People with cancer may experience a variety of emotions and reactions to their new reality that may be mild or intense, transitory or permanent.

**Objectives:**

The aim of our study was to assess the psychological distress of patients with breast cancer.

**Methods:**

Cross-sectional descriptive study including patients followed for breast cancer at the outpatient medical oncology consultation of Hadj Ali Soua regional hospital from January to March 2021. We used the “Hospital Anxiety and Depression Scale (HAD-S)” for the assessment of anxiety and depression.

**Results:**

Fifteen patients were included with a mean age of 49.87 ± 8.48 years and a mean age at diagnosis of 46.73 ± 7.55 years. At the TNM classification, 66.6% of the patients had a T1 or T2 at the time of diagnosis and 80% had an N0. All patients received a surgical intervention which was conservative in 53.3% of cases. No patient underwent breast reconstruction. Chemotherapy and hormone therapy were prescribed in 86.7% of patients. The mean anxiety and depression scores according to the HAD-S were 9.53 and 4.93, respectively. The majority of our patients had no depressive symptoms (80%) against only 2 patients (13.33%) with depressive symptoms. On the other hand, most of our patients were anxious: 6 patients (40%) showed probably clinically relevant levels of anxiety (score of 11 or higher) and 5 patients showed possibly clinically relevant levels of anxiety (scores of 8 or higher) (33,33%).

**Conclusions:**

Our study revealed a high prevalence of psychological distress. The presence of clinical psychologists in the medical oncology department and the training of nursing staff in psycho-oncology are essential for the overall care of patients with cancer.

**Disclosure of Interest:**

None Declared

